# Prevention of Radiation-Induced Bladder Injury: A Murine Study Using Captopril

**DOI:** 10.1016/j.ijrobp.2022.10.033

**Published:** 2022-11-16

**Authors:** Angela M. Groves, Nicole Paris, Eric Hernady, Carl J. Johnston, Omar Aljitawi, Yi-Fen Lee, Sarah L. Kerns, Brian Marples

**Affiliations:** * Departments of Radiation Oncology, University of Rochester, Rochester, New York; † Departments of Pediatrics, University of Rochester, Rochester, New York; ‡ Departments of Medicine, Hematology/Oncology, University of Rochester, Rochester, New York; § Departments of Urology, University of Rochester, Rochester, New York

## Abstract

**Purpose:**

Pelvic radiation therapy (RT) can cause debilitating bladder toxicities but few clinical interventions exist to prevent injury or alleviate symptoms. From a large genome-wide association study in patients with prostate cancer it was previously reported that SNPs tagging *AGT*, part of the renin-angiotensin system (RAS), correlated with patient-reported late hematuria, identifying a potential targetable pathway to prevent RT-induced bladder injury. To investigate this association, we performed a preclinical study to determine whether RAS modulation protected the bladder against RT injury.

**Methods and Materials:**

C57BL/6 male mice were treated with an oral angiotensin converting enzyme inhibitor (ACEi: 0.3g/L captopril) 5 days before focal bladder X-irradiation with either single dose (SD) 30 Gy or 3 fractions of 8 Gy (8 Gy × 3 in 5 days). RT was delivered using XStrahl SARRP Muriplan CT-image guidance with parallel-opposed lateral beams. ACEi was maintained for 20 weeks post RT. Bladder toxicity was assessed using assays to identify local injury that included urinalysis, functional micturition, bladder-released exosomes, and histopathology, as well as an assessment of systemic changes in inflammatory-mediated circulating immune cells.

**Results:**

SD and fractionated RT increased urinary frequency and reduced the volume of individual voids at >14 weeks, but not at 4 weeks, compared with nonirradiated animals. Urothelial layer width was positively correlated with mean volume of individual voids (*P* = .0428) and negatively correlated with number of voids (*P* = .028), relating urothelial thinning to changes in RT-mediated bladder dysfunction. These chronic RT-induced changes in micturition patterns were prevented by captopril treatment. Focal bladder irradiation significantly increased the mean particle count of urine extracellular vesicles and the monocyte and neutrophil chemokines CCL2 and MIP-2, and the proportions of circulating inflammatory-mediated neutrophils and monocytes, which was also prevented by captopril. Exploratory transcriptomic analysis of bladder tissue implicated inflammatory and erythropoietic pathways.

**Conclusions:**

This study demonstrated that systemic modulation of the RAS protected against and alleviated RT-induced late bladder injury but larger confirmatory studies are needed.

## Introduction

Late radiation cystitis (RC) can be a severe debilitating complication after pelvic radiation therapy (RT).^1–3^ Radiation-mediated tissue inflammation and fibrosis contribute to the development of RC and other adverse RT-associated bladder dysfunctions, but the defining molecular mechanisms are not well described. There are no durable therapies to prevent the development of RC.^4^ Hyperbaric oxygen therapy (HBOT) does alleviate the symptoms of RC once established^5,6^; however, HBOT is not widely available, is contraindicated in some patients, and is cost-prohibitive, which limits its overall clinical utility.

A genome-wide association study (GWAS) among 3871 patients with prostate cancer receiving RT identified SNPs, tagging *AGT*, correlated with gross hematuria, which is a defining symptom of RC,^7^ and pointed to a targetable pathway to prevent RC. *AGT* encodes angiotensinogen, part of the renin-angiotensin system (RAS), and precursor of Ang I. Angiotensin-converting enzyme (ACE) cleaves Ang I to biologically active Ang II, which on binding with its receptor (AT1R) regulates tissue inflammation.^8–11^ In a separate clinical cohort study, RC was seen in only 4.8% of patients with prostate cancer taking an ACE inhibitor (ACEi) during RT compared with 16.5% not taking an ACEi (*P* = .01; hazard ratio adjusted for clinical risk factors = 0.51, 95% CI 0.28-0.94), indicating that inhibition of ACE could protect against RT-induced bladder injury.^12^

Captopril is a well-tolerated FDA-approved RAS ACEi previously shown to mitigate chronic radiation-induced injury in other normal tissues.^13,14^ To investigate whether an ACEi could prevent and mitigate the development of radiation-induced bladder dysfunction, C57BL/6 male mice were given oral captopril 5 days before focal bladder CT-image-guided X-irradiation and bladder function was assessed for the next 20 weeks. This dosing regimen was selected to mimic ACEi use in a retrospective human study. In this study, we report that focal bladder RT altered normal bladder function and that long-term RAS modulation corresponded with preserved micturition patterns and bladder histology, and reductions in circulating inflammatory-mediated immune cells. These data demonstrate for the first time that use of RAS modulation can prevent radiation-induced bladder dysfunction in a murine model.

## Methods and Materials

### Animals

All animal protocols were approved by the Institutional Animal Care and Use Committee at the University of Rochester and conform to National Institutes of Health guidelines. Male C57BL/6 mice (6-8 weeks of age, 22-26 g) were purchased from The Jackson Laboratory (Bar Harbor, Maine). Mice were randomized into the various treatment groups, and initially housed 5 to a cage with access to food (standard laboratory chow) and water ad libitum. Mice were weighed weekly. Captopril (Alfa Aesar, Ward Hill, Massachusetts), dissolved in drinking water at a dose of 0.3g/L, was administered starting 5 days before irradiation and maintained throughout the course of the experiment.

### Radiation treatment

Mice were irradiated at room temperature using CT-guidance with an XStrahl SARRP X-irradiator (Small Animal Radiation Research Platform; XStrahl Inc, Suwanee, Georgia; 220kVp/13.0mA at a dose rate of 3.1 Gy/min). Visipaque contrast agent (GE Health care, 270 mg/mL, Marlborough, Massachusetts; 25-50g/L) was delivered via retro-orbital injection to identify the bladder and animals were irradiated in a supine position to aid bladder targeting. Isoflurane anesthesia (1-3% with balance oxygen) was used for immobilization. The SARRP Muriplan program was used to deliver an opposing 2-beam set-up with beam collimation using a variable collimator to ensure complete bladder coverage. Beams were positioned in a transverse orientation to avoid off-target tissue exposure. Dose volume histograms (DVHs) were used to confirm no dose was delivered to off-target tissue. Control animals were sham irradiated with identical handling. A single dose of 30 Gy or 8 Gy × 3 (BED_3Gy_ = 88 Gy) were used with n = 5 animals per treatment cohort. The fractionated dosing regimen was less intense than a conventional clinical dosing regimen of 35 fractions of 2 Gy (BED_3Gy_ = 116.6 Gy).

### Hyperbaric oxygen therapy

A self-designed and constructed HBOT chamber was used to administer a single 90-minute course of HBOT. Activated charcoal was placed in the chamber for CO_2_ absorption before initiation of the compression cycle. The chamber was raised to 100% oxygen over 2 minutes, and then pressure increased to 2 atm at a rate of 2 psi/min, and maintained for 90 minutes. After the HBOT, the chamber was decompressed at a rate of 2 psi/min (over 18 minutes) to background oxygen levels and the mice were removed to regular housing.

### Sample collection

Micturition parameters of void volume and voiding frequency were measured using a void spot assay.^15,16^ Assays were performed at the same time of day during the light cycle. Individual mice were placed in a regular mouse cage with the entire base of the cage completely lined with What-man grade filter paper. Mice were undisturbed and allowed to urinate freely for a 2-hour observation period, during which access to food and water was restricted. Filters were then imaged with UV light and photographed for image analysis. Images were processed using ImagePro Plus software (version 7.0). Areas of interest (AOI) were designated from the raw images to delineate individual voids on the filter paper and then binary image masks were constructed for each void. The area of each individual discrete void and the number of individual voids on each filter paper were then quantified for each individual mouse. Objects less than 300 pixels were excluded from the analysis to eliminate recording paw prints and tail dragging marks. For micturition analysis, discrete voiding events were binned into 3 different categories based on size, to represent large pools (10,000+ pixels), large voids (9999-900 pixels) and small voids (899-300 pixels). The number and volume of individual voids were calculated for each individual mouse (ie, each individual filter paper) in each experimental condition. Then, the mean number and volume of voids were calculated from these values for each treatment condition from all the mice in that cohort. Consequently, individual mice were treated as discrete entities and averaged within each group at each time point. The number of voids per filter paper in each size category were also calculated. This process was repeated to longitudinally assess micturition for each mouse throughout the course of the study.

### Urinalysis

Urine was collected throughout the course of the study by gently scruffing the mouse to induce voiding. Several drops of expelled urine were collected on a glass plate. Urine was then applied to urine test strips (Chemstrip 10 with SG; Roche Diagnostics, Indianapolis, IN) and evaluated. Values for each mouse were then treated as discrete entities and averaged within each group at each time point.

### Complete blood counts

Submandibular bleeds were performed on mice throughout the duration of the study at a frequency of no less than 1 month apart, and blood was collected into EDTA containing purple top microtainer tubes (Becton, Dickinson and Company, Franklin Lakes, New Jersey). Samples were analyzed using a HESKA Hematrue instrument (HESKA Corporation, Loveland, Colorado).

### ELISA

At the time of euthanization and bladder collection, whole blood was drawn via cardiac puncture into EDTA-containing purple top microtainer tubes (Becton, Dickinson) and after centrifugation plasma was collected, then aliquoted and stored at −85° C. Sandwich enzyme immunoassay kits (R&D Systems, Minneapolis, Minnesota), performed according to the manufacturer’s instructions, were used to quantitatively measure plasma levels of erythropoietin (Mouse Erythropoietin Quantikine ELISA, Catalog Number MEP00B).

### Histology

Bladders were emptied of urine at time of collection. The bladder was fixed by immersion in zinc-buffered formalin (AnaTech, Battle Creek, Michigan) for 16 to 24 hours with the bladder neck open. Then the tissues were dehydrated using graded alcohols (Ultrapure, North Darien, Connecticut) and cleared through several changes of xylene (VWR International, West Chester, Pennsylvania). Bladders were embedded in paraffin, sectioned at 5 *μ*m, and mounted on Colormark Plus microscope slides (Thermo Scientific, Portsmouth, New Hampshire). Collagen staining was achieved using Gomori’s trichrome (Gomori Trichrome Stain Kit, Leica Biosystems Richmond, Inc, Richmond, Illinois). Picro Sirius Red staining was performed using Polysciences Red Stain kit (Polyscience, Inc, Cat#24901-250). Brightfield images at a 20 × magnification were acquired using an Olympus BX51 microscope (Olympus America, Center Valley, Pennsylvania) fitted with a Spot Pursuit camera (Diagnostic Instruments, Inc, Sterling Heights, Michigan).

### Quantification of urothelium width

For each Gomori’s trichrome stained specimen, the urothelial layer was imaged in at least 3 fields. Images were processed using ImagePro Plus software. For each bladder, 2 to 4 fields were imaged, at least 30 vectors were drawn across the width of the urothelium within each field, and a single value representing the average width of each specimen was calculated. These single values were then used to determine the average width for each experimental condition.

### Quantification of collagen fiber integrity and density

Each Picro Sirius Red-stained specimen was imaged in at least 3 fields, each with and without polarized light, and brightfield images were acquired as described previously. For collagen fiber analysis, ImagePro Plus software was used to threshold pixels above a designated intensity and determine percentage above threshold. Fiji Is Just ImageJ (FIJI) was used to measure integrated density of the threshold images.

### Transcriptomics

Total RNA was isolated using the Zymo Direct-zol Mini RNA kit (Irvine, California), concentration was determined via NanoDrop, and RNA quality assessed with the Agilent Bioanalyzer (Agilent, Santa Clara, California). Total RNA sequencing was performed using the TruSeq Stranded mRNA Sample Preparation Kit (Illumina, San Diego, California) and single end reads of 100 bp were generated for each sample using Illumina’s NovaSeq 6000 sequencer. Raw reads were demultiplexed using bcl2fastq version 2.19.0. quality filtering and adapter removal using FastP version 0.20.0; processed/cleaned reads were mapped to the GRCm38 + gencode M22 reference using STAR_2.7.0f. Gene level read quantification was derived using the subread-1.6.4 package (featureCounts) and differential expression analysis was performed using DESeq2-1.22.1 with a *P* value threshold of 0.05 within R version 3.5.1 (https://www.r-project.org/). Gene set enrichment used Enrichr^17–19^ with a list of differentially expressed genes at the nominal *P* value cut-off 0.05 as input for each comparison. Reported *P* values were adjusted for multiple testing using the Benjamini-Hochberg false discovery rate method.

### Statistics

GraphPad Prism 9.3.1 software was used for the statistical analyses (GraphPad Software, LLC, San Diego, California). Data are presented as mean ± SEM. Statistical significance was determined by 2-way ANOVA followed by Tukey’s Multiple Comparison Test when ANOVA showed significant differences. A value of *P* < .05 was considered significant. For the correlation of micturition parameters with urothelial width, the data points are measures for average values of volume and number of voids calculated for individual mice plotted against the average vector length per specimen of the corresponding mouse. Spearman correlations and the resulting r and *P* values are reported.

### Exosome analysis

Urine samples were collected from C57BL/6 mice treated with focal bladder RT ± captopril. Extracellular vesicles (EVs) were purified and particle numbers and size distribution analyzed using Nanoparticle Tracking Analysis (NTA), as per published methods.^20–25^

## Results

**Fig. 1.**
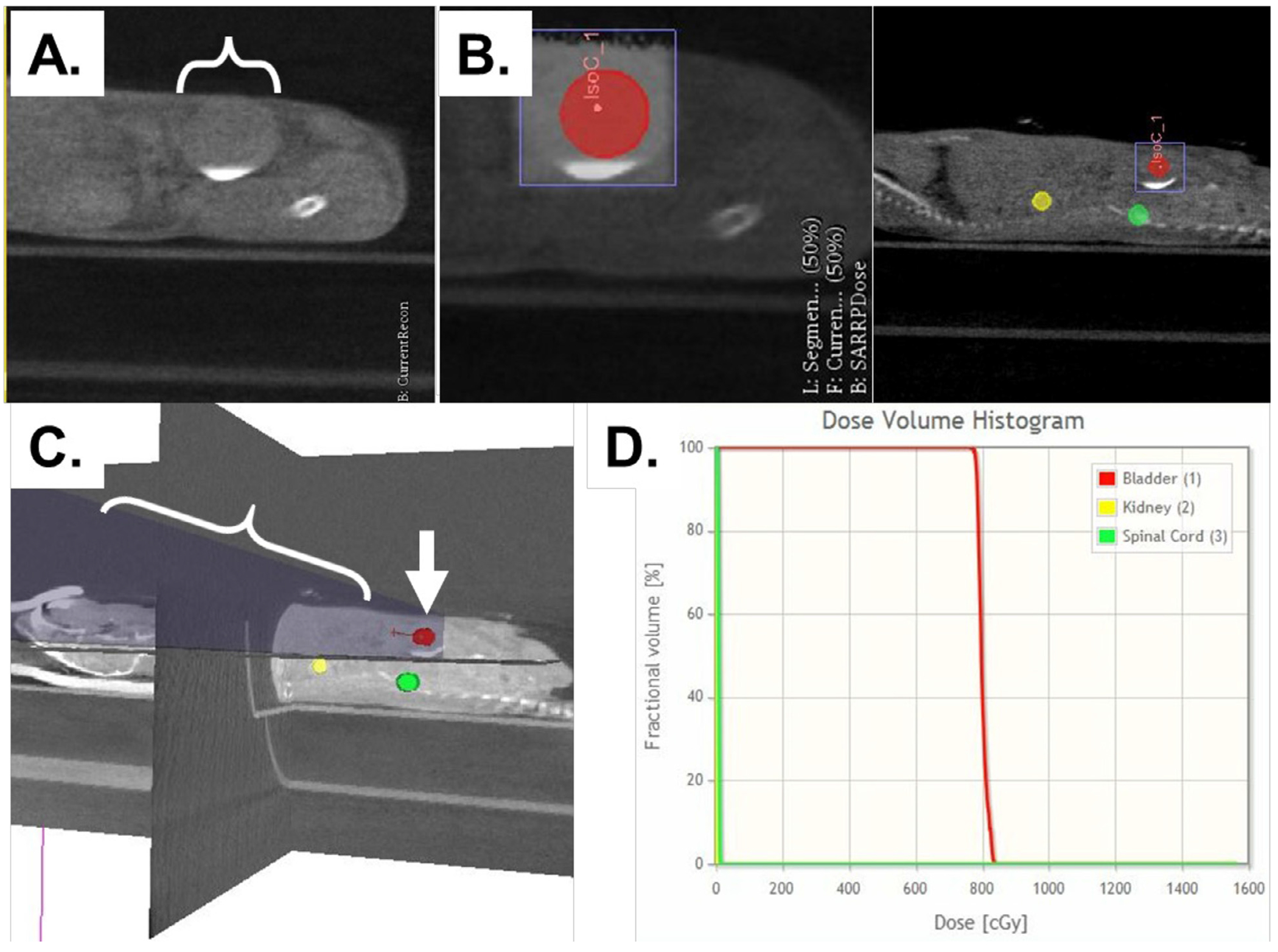
Animal placement (supine), treatment planning, and focal bladder irradiation using the Small Animal Radiation Research Platform x-ray unit and treatment planning. (A) Visipaque identifying the bladder (beneath white bracket). (B) Placement of bladder-targeted isocenter (red; IsoC_1) and radiation field; the volume of tissue irradiated is shown by the purple square. (C) X-ray beam set-up (transverse, blue column indicated by bracket, bladder identified by white arrow) and dosing. (D) Dose volume histogram shows 100% of the dose targeted to the bladder. Off-target tissue, kidney, and spine (yellow and green circles, respectively, in panel B) show no dose.

Radiation treatments were individually customized for each mouse using the SARRP MuriPlan dose planning system. A variable beam collimator was used to ensure full-volume bladder coverage. Figure 1 shows a typical DVH plan for the targeted bladder and off-target kidney and spinal cord, and demonstrates the whole volume bladder received the required dose with negligible off-targeting dosing. Urine and blood were longitudinally harvested, starting before irradiation until euthanization, and functional micturition assays were performed at 4 and 15 weeks. Body weight (Fig. E1) was used to assess animal health.

**Fig. 2.**
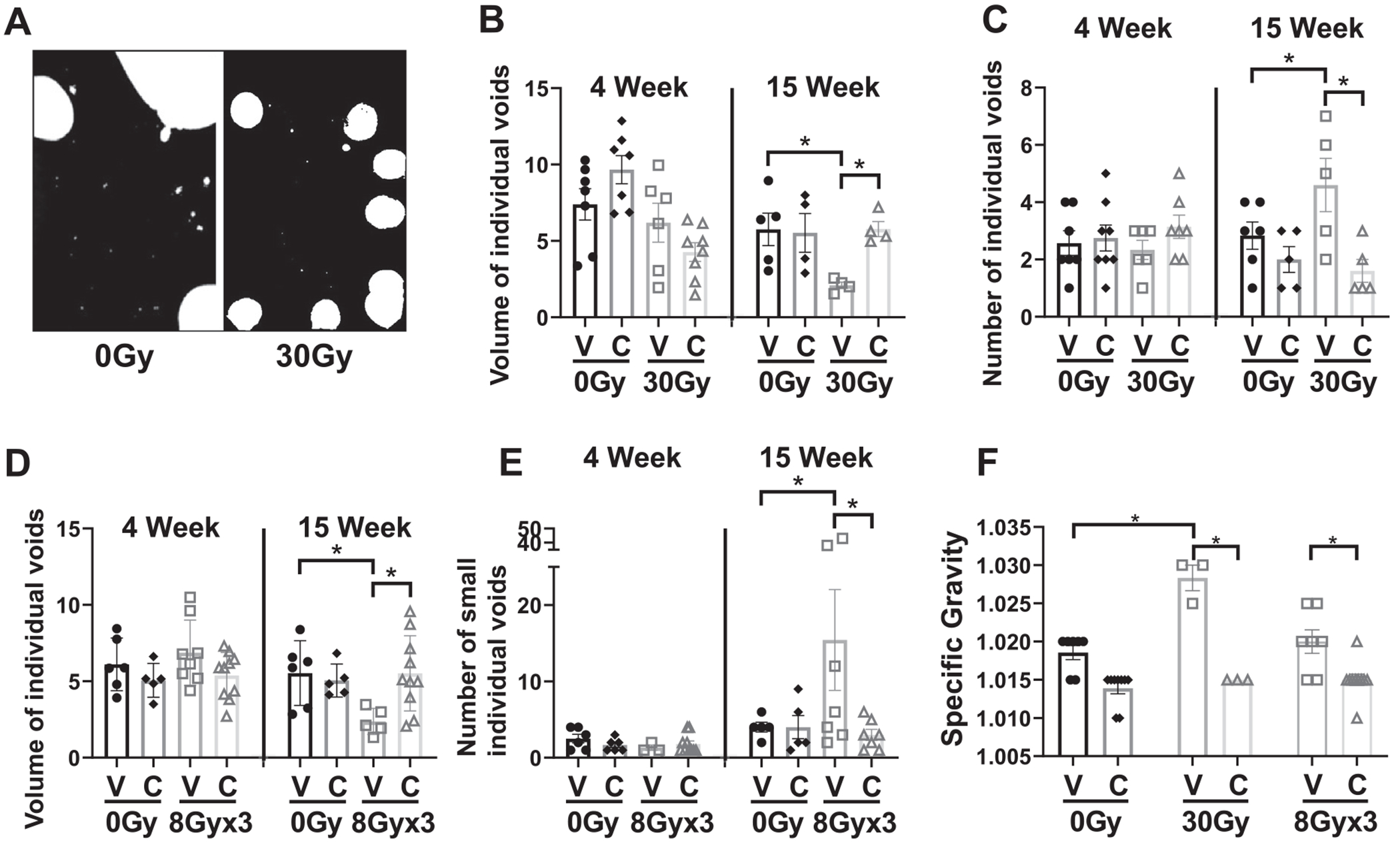
Assessment of bladder function. (A) Micturition evaluation: pattern of urination showing individual voids and volume of each void measured over a 2-hour period for a single mouse irradiated with 30 Gy and a nonirradiated mouse. (B) Mean volume of individual voids and (C) number of individual voids were determined from averages of values calculated for individual mice (ie, from a single filter paper) exposed to either 30 Gy focal bladder irradiation or to 0 Gy control, and treated with either captopril (C) or vehicle (V), at 4 weeks (n = 6-8) and 15 weeks (n = 4-5) after irradiation. (D) Mean volume of individual voids (large pools >10,000+ pixels and large voids >900 pixels) and (E) number of small individual voids less than 900 pixels were determined from averages of values calculated for individual mice exposed to 8 Gy × 3 or 0 Gy treated with either captopril (C) or vehicle (V), at 4 weeks (n = 5-9) or 15 weeks (n = 5-10) after irradiation. (F) Specific gravity of urine collected at 10 weeks (for 30 Gy exposure) or 15 weeks (for 8 Gy × 3 exposure) or to 0 Gy.

Figure 2 shows acute and chronic radiation-induced changes in micturition, expressed as the number and volume of individual bladder voids for each mouse over a 2-hour period, averaged for each experimental condition. Micturition changes were assessed by measuring the number and volume of all individual urination events. A typical example, shown in Fig. 2A, illustrates an increased number of individual voiding events (from 3 to 7) with a reduced volume per the individual void when comparing 2 aged-matched mice, 1 of whom was treated with 30 Gy bladder-targeted irradiation and the other not irradiated.

For nonirradiated mice (0 Gy), captopril or vehicle treatment alone did not significantly increase or decrease the number or volume of individual voids (Figs. 2B, C). Also, no significant changes in micturition were seen for any treatment conditions at the acute 4-week timepoint (Figs. 2B, C). However, at 15 weeks, the 30 Gy + vehicle treatment caused a significant decrease in the mean volume of individual voids calculated from all mice in the cohort compared with the mean of 0 Gy + vehicle mice (Fig. 2B, right panel): down from 5.55 ± 0.83 at 0 Gy + vehicle to 2.16 ± 0.46 at 30 Gy + vehicle (*P* = .0366), while the mean number of voids significantly increased (from 2.83 ± 0.58 at 0 Gy + vehicle to 4.25 ± 0.85 at 30 Gy + vehicle, *P* = .0326 Fig. 2C, right panel). Treatment with captopril prevented the decrease in mean void volume that resulted after 30 Gy (2.16 ± 0.46 after 30 Gy + vehicle vs 5.9 ± 0.87 after 30 Gy + captopril, *P* = .0429). Treatment with captopril also prevented the increase in number of individual voids (4.25 ± 0.85 at 30 Gy + vehicle reduced to 2.33 ± 0.98 at 30 Gy + captropril, *P* = .0128, Fig. 2C).

For mice treated with 8 Gy × 3, no changes in the volume of individual voids or number of small voids were seen at 4 weeks, with or without captopril (Figs. 2D, E). In contrast, at the 15-week timepoint, the mean volume of individual voids decreased after 8 Gy × 3 alone (Fig. 2D, right panel): down from 5.54 ± 0.86 at 0 Gy + vehicle to 2.37 ± 0.38 at 8 Gy × 3 + vehicle (*P* = .023); and this was associated with an increase in the mean number of small-volume voids (Fig. 2E, right panel) although there was a wide variation within the data set. Captopril treatment prevented the significant reduction in void volume seen after irradiation alone: 2.37 ± 0.38 for 8 Gy × 3 + vehicle versus 5.51 ± 0.78 for 8 Gy × 3 + captopril (*P* = .042), as well as number of individual voids. Captopril treatment also prevented the significant increase in urine specific gravity, a measure of the number of particles in urine and density of urine, at 10 and 15 weeks compared with mice treated with 30 Gy or 8 Gy × 3 alone (Fig. 2F).

**Fig. 3.**
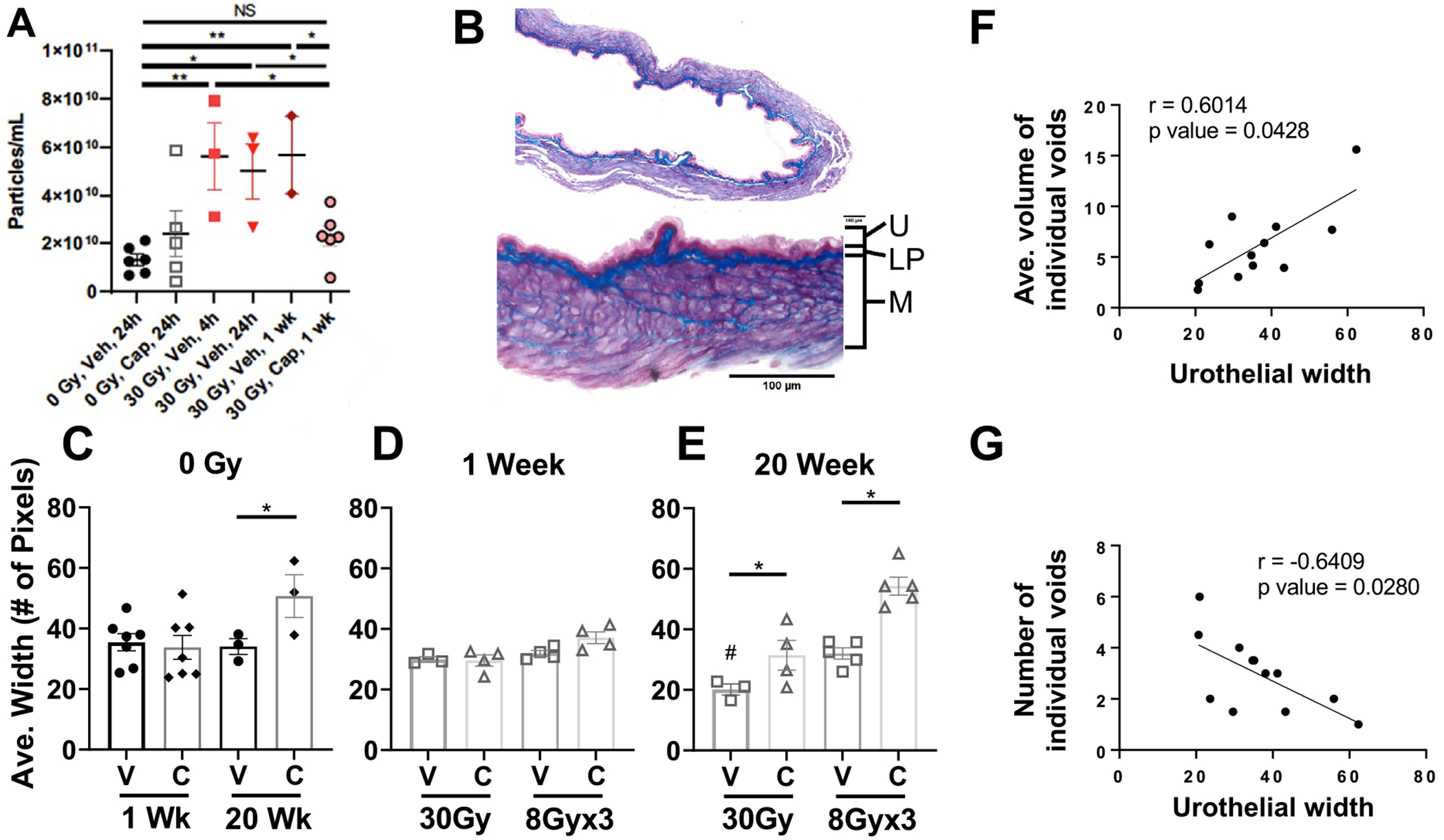
Exosome analysis and histopathology of bladder from mice exposed to either 30 Gy or 8 Gy × 3 targeted to bladder or to 0 Gy control and treated with either captopril (Cap), or vehicle (Veh) at 4 hours to1 week after irradiation. (A) Exosome particle size in urine after 30 Gy ± captopril (means ± SEM). *P < .05, **P < .01 (*P* values were not corrected for multiple comparisons given that the experiments were preliminary in nature because of small sample sizes). (B) Representative image of Gomori Trichrome stained bladder in an untreated mouse, depicting urothelium (U), lamina propria (LP), and detrusor smooth muscle (M). Images of urothelium were used for analysis of urothelial width. Effects of bladder irradiation and captopril treatment on average thickness of urothelial layer in (C) 0 Gy control mice, (D) bladder irradiated mice at 1 week, and (E) at 20 weeks: n = 3 to 6 mice/treatment group. Urothelial layer thickness correlates with micturition parameters of (F) volume of individual voids and (G) number of voids/filter paper in 30 Gy mice. n = 12 mice for correlation studies.

The specific gravity data was expanded by measuring the numbers of extracellular vesicles (EVs; eg, exosomes and microvesicles) in mouse urine (Fig. 3A) because EVs shed by irradiated cells have been linked to radiotoxicity and the regulation of inflammatory responses^26,27^ and can also serve as alternate carriers for the delivery of cytokines.^28,29^ EVs are important vehicles in mediating fibrotic pathogenesis.^30^ This analysis demonstrated that captopril alone with no irradiation (0 Gy) did not significantly change the number of EVs in urine compared with 0 Gy + vehicle. However, treatment with 30 Gy significantly increased the number of EVs at 4 hours, 24 hours, and 1week post RT, albeit these studies need additional validation. Captopril prevented the increase in EVs seen after 30 Gy.

Animals were euthanized at 1 week and 20 weeks, to assess tissue inflammation and fibrosis respectively, after SD and fractionated radiation alone, and radiation plus captopril treatments by assessing changes to the width of the bladder urothelium and integrity of the detrusor muscle (Fig. 3B, representative 0 Gy image). Animals irradiated with either 30 Gy or 8 Gy × 3 without captopril showed no change in urothelial width at 1 week (Fig. 3D) but significant changes were seen at 20 weeks (Fig. 3E). Captopril treatment alone did not affect the urothelial width for nonirradiated control animals at 1 week (Fig. 3C) although it did increase urothelial width in nonirradiated animals after 20 weeks of treatment. In animals treated with SD 30 Gy, captopril treatment (34.96 ± 1.65) significantly prevented urothelial thinning at 20 weeks compared with 30 Gy + vehicle (20.85 ± 1.97); *P* = .027. Similar observations were seen for animals treated with 8 Gy × 3: captopril treatment (54.26 ± 4.32) significantly prevented urothelial thinning compared with 8 Gy × 3 + vehicle (35.15 ± 1.87); *P* = .002 (Fig. 3E). Urothelial width was positively correlated with mean volume of individual voids (r = 0.6014; *P* = .0428) suggesting urothelial thinning was associated with smaller volume voids, and because the number of voids was negatively correlated with urothelial width (r = −0.6409; *P* = .028) then thinning of the urothelial layers suggests more frequent voiding (Fig. 3F, 3G). To assess changes in detrusor muscle, the organization of collagen in the muscle after irradiation along with collagen deposition was preliminarily investigated after 30 Gy. Irradiation induced changes in collagen organization and deposition and this was alleviated by captopril treatment (Fig. E2).

Captopril was administered orally, and as such, its protective effects on micturition might result from local effects in the irradiated bladder or from indirect systemic modulation of the renin-angiotensin system. To explore the role of local inflammation, molecular signaling was assessed at 4 hours and 1 week after RT using RNAseq to assess transcriptome changes in whole-bladder tissue. As expected, a robust transcriptional response to radiation was seen when comparing bladders from irradiated mice with nonirradiated controls: 9914 genes differentially expressed (out of 55,453 measured, 17.9%) at 4 hours and 2980 genes differentially expressed (5.4%) at 1 week after 30 Gy compared with nonirradiated age-matched control mice. This transcriptional response to radiation was tempered when mice were treated with the captopril: 7000 genes differentially expressed (12.6%) at 4 hours and 1991 genes differentially expressed (3.6%) at 1 week. TNF-alpha signaling via NF-kB pathway of the MSigDB Hallmark database was enriched for differentially expressed genes at 1-week post-RT compared with no RT (adjusted *P* = 7.15 × 10^−5^), and this pathway was no longer enriched when mice were treated with captopril (adjusted *P* = .30). These database studies indicate that captopril modulates inflammatory processes that occur in response to RT (Table E1). In agreement with this observation, it was observed that several inflammatory pathways were enriched for differentially expressed genes 1 week after RT when comparing mice treated with captopril with mice receiving vehicle (eg, TNF-alpha Signaling via NF-kB; IL-2/STAT5 Signaling; Inflammatory Response; IL-6/JAK/STAT3 Signaling; Interferon Gamma Response). We were particularly interested in the EPO pathway because EPO expression is regulated by Angiotensin II,^31,32^ and because this pathway is not represented in MSigDB, we queried the BioCarta database. It was also found that the BioCarta erythropoietin (EPO) pathway was enriched for differentially expressed genes when comparing RT to no RT among mice treated with captopril. This was not unexpected as the angiotensin-converting enzyme captopril reduces the conversion of Angiotensin I to Angiotensin II, and this would correspondingly alter EPO levels.

**Fig. 4.**
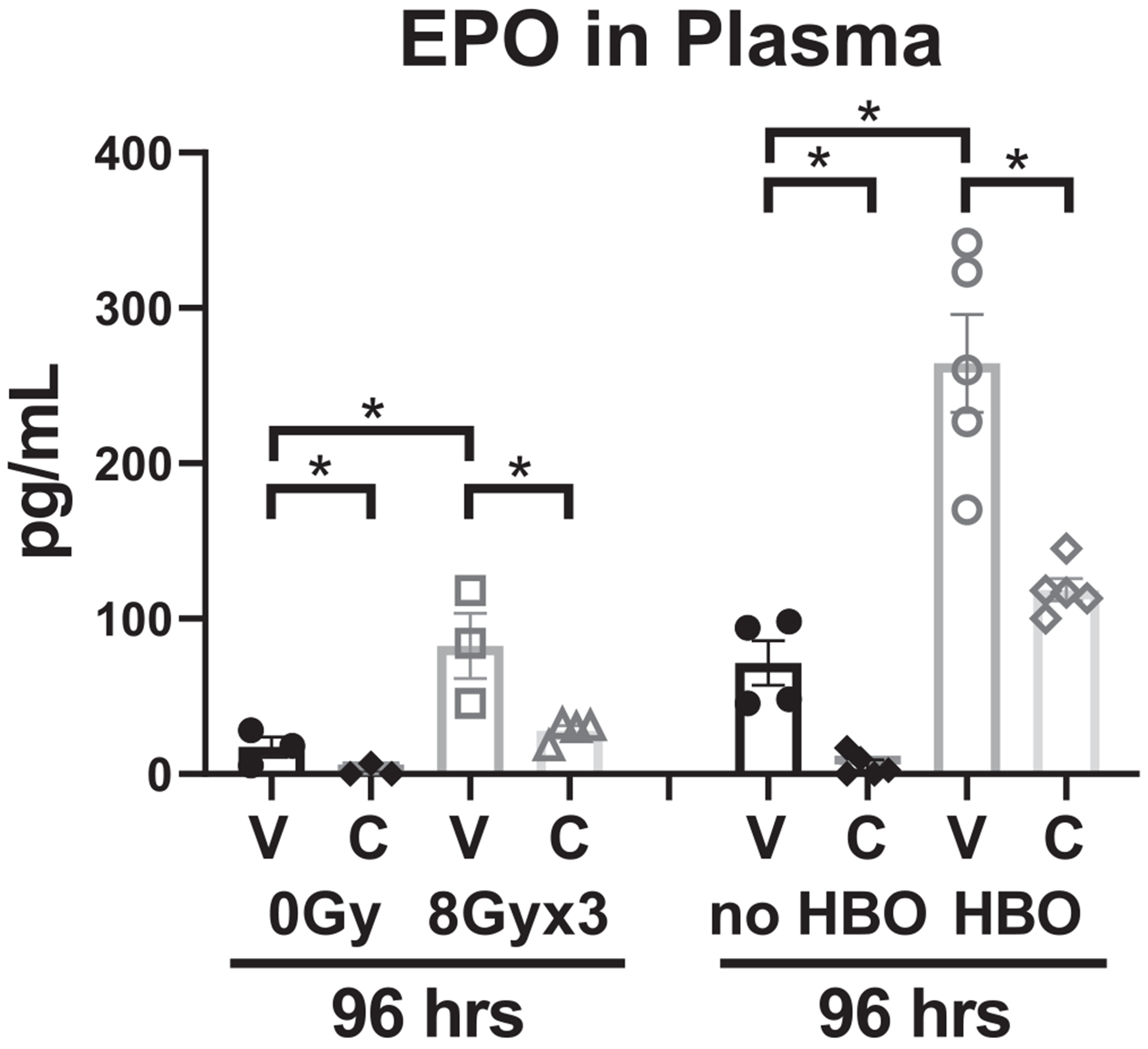
Erythropoietin (EPO) concentration in plasma, collected from mice exposed to either 8 Gy × 3 targeted to bladder or to 0 Gy control, as well as mice exposed to hyperbaric oxygen (HBO) as a positive control for EPO production and treated with either captopril (C), or vehicle (V). Blood was collected at 96 hours after the first fraction of irradiation, or at 96 hours after completion of HBO treatment. n = 3 to 5 mice/treatment group. *P < .05.

To further investigate the systemic modulation of EPO expression by captopril treatment, as a noninvasive direct surrogate marker of Angiotensin II expression, changes in EPO expression in serum 96 hours after 8 Gy × 3 treatment regimen ± captopril were assessed, and HBO treatment was included as a positive control for EPO expression (Fig. 4). It was observed that captopril reduced EPO expression in irradiated and HBO-treated animals, confirming captopril treatment systemically indirectly reduced levels of Angiotensin II.

**Fig. 5.**
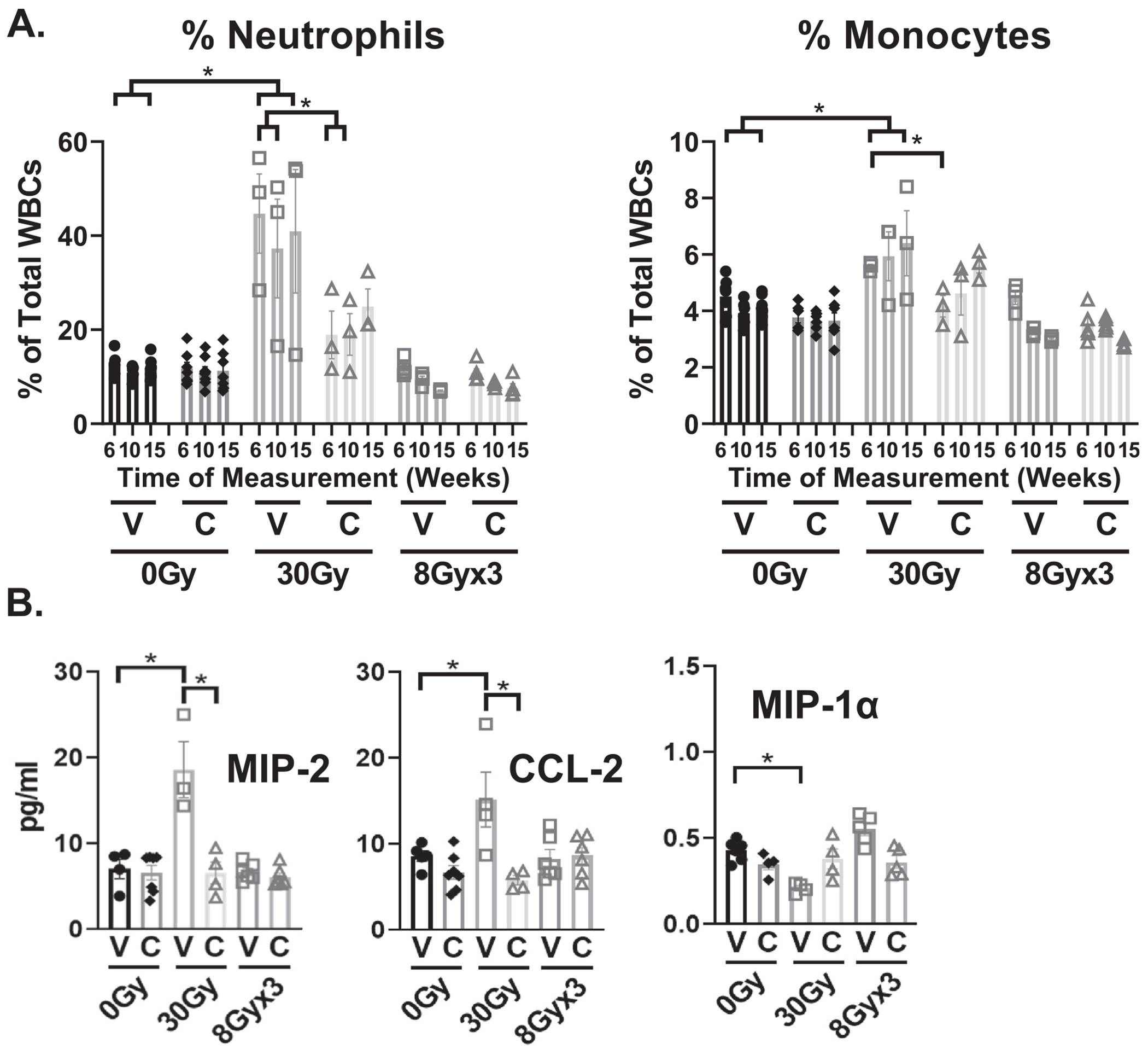
(A) Proportions of circulating neutrophils and monocytes collected in whole blood from mice exposed to either 30 Gy or 8 Gy × 3, targeted to bladder, or to 0 Gy control and treated with either captopril (C), or vehicle (V), at 6, 10, and 15 weeks following irradiation. (B) CCL-2, MIP-1*α*, and MIP-2 concentration in plasma analyzed by ELISA at 1week after either SD 30 Gy or 8 Gy × 3. n = 3 to 6 mice/treatment group. *P < .05. *Abbreviations:* CCL-2 = C-C motif chemokine ligand 2, MIP-1*α* = macrophage inflammatory protein 1*α*, MIP-2 = macrophage inflammatory protein 2.

To investigate the consequences of local tissue inflammation, changes in circulating neutrophils and monocytes were assessed because these cell populations respond to radiation-induced tissue inflammation. As expected, after 30 Gy focal bladder irradiation, the proportion of circulating neutrophils and monocytes within the white blood cell (WBC) count significantly increased (Fig. 5), while they were persistently and significantly reduced with captopril treatment 6 to 15 weeks after irradiation. Captopril administration alone did not have any significant effects on the proportion of circulating cells in *nonirradiated* animals (Fig. 5A, 0 Gy + V compared with 0 Gy + C), demonstrating that bladder irradiation was needed to elicit the reduction in neutrophils and monocytes. These changes in circulating immune cells were associated with changes in the levels of circulating chemokines that recruit monocytes and neutrophils, and these were also alleviated by captopril treatment (eg, CCL-2, MIP-1α, and MIP-2, Fig. 5). Taken together, these data demonstrate that systemic administration of captopril can modulate systemic radiobiological responses after local bladder irradiation. The changes in circulating neutrophils and monocytes evident after 30 Gy were less prominent after 8 Gy × 3, and elevated levels were seen only after 6 weeks with a response that did not persist.

## Discussion

RT-associated bladder toxicities are a clinical concern for patients treated for prostate, cervix, and bladder cancer, among others. Bladder toxicity is notably reported after various prostatic RT treatment regimens,^33–35^ and gross hematuria is 1 significant recurring toxicity, with 5-year and 10-year incidence rates ranging from 5% to 18%.^36,37^ More generalized adverse radiation-mediated bladder effects include micturition frequency, nocturia, dysuria and incontinence. All are bothersome, and when severe can necessitate invasive procedures with additional associated risks.^1,6^

A GWAS in 6 large prostate cancer RT cohorts identified a potentially targetable pathway because SNPs tagging *AGT* correlated with patient-reported hematuria.^7^ To investigate the relationship between *AGT* and the RAS for regulating radiation-induced bladder toxicity, a murine model was used to determine whether modulating the RAS was radioprotective. The use of captopril preserved normal urinary function (defined as maintenance of urination frequency and volume of each individual void) and the avoidance of urinary dysfunction attributed to radiation-mediated bladder injury. These data also demonstrated that urinary dysfunction was positively correlated with the width of the bladder endothelium at 20 weeks post RT (ie, radiation-mediated thinning of the urothelium at 20 weeks was linked with more frequent urination) and this was ameliorated by captopril. Moreover, the volume of individual voids was correlated with urothelium width (ie, radiation-mediated thinning of the urothelium at 20 weeks was linked with smaller void volumes). The change in urothelial width seems unlikely to be an age-related change because no increase was seen in animals not given captopril. Rather, taken together, these correlations suggest the radiation-induced thinning of the urothelial layer was associated with more frequent and smaller volume voiding. Captopril use did not provide acute protection of the urothelium 4 weeks after irradiation; instead, it protected against late effects, which is consistent with a GWAS and clinical cohort study.^12^ The current results demonstrated a temporal nature to the effects of captopril on RT-induced bladder dysfunction and inflammation. Although it was found that chronic/late functional changes were ultimately tempered by captopril, no early changes in functional micturition with or without captopril were seen, despite evidence of changes in early/acute inflammation (ie, circulating proinflammatory chemokines and immune cells, and upregulation of proinflammatory transcriptional pathways). However, these early/acute inflammatory changes were alleviated by ACE inhibition, indicating that captopril-modulating events in tissue begin before the appearance of functional deficits. These data support our hypothesis that acute injury dictates the severity of late effects, and the more extensive acute injury to the urothelium prompts earlier and more extensive chronic injury. We hypothesize that RT injury, occurring principally in the urothelium at the outset, prompts the initial inflammatory-mediated acute toxicities, which then contribute to the development of chronic profibrotic mechanisms in the muscular layer. Fibrosis develops progressively because of a profibrotic microenvironment causing collagen deposition and dysfunction, events that reduce bladder compliance and result in the observed late functional impairments. Loss of the epithelium produces continuous irritability of the bladder wall from exposure to urine^38^ and likely contributes to some the correlative effects we saw.

Previous studies using female C3H mice treated with SD 12 to 29 Gy bladder irradiation revealed that the acute phase of bladder injury consisted of 2 waves of injury, from 1 to 15 days and 16 to 30 days,^39,40^ and indicated the dose at which 50% of the irradiated animals showed a 50% reduction in bladder capacity between the 2 waves was ~20 Gy. Subsequent studies using SARRP-targeted focal bladder irradiation using female C3H mice treated with SD 20 Gy^38^ or female C57BL/6 mice with SD 40 Gy^41^ have demonstrated loss of E-cadherin, ZO-1, and Uroplakin III^41^ and damage to the barrier function of small subepithelial blood vessels^42^ underlaying long-term urothelial integrity of the bladder. The current study using male C57BL/6 mice treated with 30 Gy and 8 Gy × 3 are consistent with these observations that urothelium injury is important in radiation-mediated bladder dysfunction.

EVs are small membrane-bound vesicles containing cargoes of proteins, lipids, and nucleotides that are secreted into the extracellular space and play various roles in physiological and pathologic processes, including response to RT,^43^ in a dose- and time-dependent manner.^44^ Circulating EV cargo molecules have been reported to predict RT efficacy^45^ and/or tissue damage in vivo as well as small-scale clinical studies.^46,47^ A significant change in mean particle count pre- to post-RT was found for animals irradiated with a single dose of 30 Gy that was prevented at 1 week by captopril treatment. Radiation-injury was associated with substantially higher post-RT particle counts. These data suggest that measuring urine EVs at the end of RT could be predictive for later-onset RT-induced bladder injury.

The acute response of the bladder to radiation injury have been attributed to impairment of the urothelial barrier and specifically the glycosaminoglycan layer, and intravesical instillation of external glycosaminoglycan restores the barrier.^48^ Previous preclinical studies using SD 20 to 40 Gy bladder-targeted irradiation demonstrated that lipo-tacrolimus instilled in the bladder,^16^ or subcutaneous injections of palifermin before irradiation,^40^ reduced radiation toxicity and mitigated functional deficits. The data described here now demonstrate that systemic oral captopril also can protect against and mitigate urinary deficits after bladder-targeted irradiation.

Mechanistically, we hypothesize the use of an ACEi prevents local accumulation of Ang II in the bladder, diminishing Ang II-AT1R signaling, and reducing vasoconstriction within the tissue. These data demonstrated that captopril treatment did reduce circulating levels of EPO, and because expression of EPO is regulated by Ang II,^31,32^ it established indirectly that captopril affects the production of Ang II. Moreover, these data suggest that a reduction in circulating EPO might be a surrogate biomarker for the efficacy of ACEi modulating Ang II and preventing radiation injury.

Ang II is known to exert proliferative and proinflammatory effects on leukocytes, endothelial cells, and vascular smooth muscle cells, and is also reported to promote profibrotic responses,^49,50^ and proinflammatory effects are mediated by increased oxidative stress. The action of Ang II is mediated through AT1 receptors, and via this interaction Ang II behaves as a vasoconstrictor and inflammatory mediator. We hypothesize that this diminution of Ang II by ACEi aids local vascular recovery in the irradiated bladder endothelium and reduces proinflammatory profibrotic signaling in the detrusor muscle. These current findings therefore provide a framework for more focused analyses on the kinetics and mechanisms of interactions between the acutely responding urothelial layer and chronically responding muscle layer.

Blockage of the AT1 receptor or use of an ACEi was sufficient for treatment of radiation-induced renal and lung injury in rodent models, and links the RAS to the pathogenesis of radiation injuries.^51,52^ This linkage is further bolstered by a prior genetic study in patients with prostate cancer supporting a role for the RAS in clinically relevant bladder toxicity.^7,12^

## Conclusion

The observation in the present preclinical model suggests that RAS modifiers such as ACEi or angiotensin receptor blockers could be a potential novel therapeutic strategy to protect against or mitigate proinflammatory and profibrotic processes that promote symptoms of bladder toxicity and radiation cystitis.

## Supplementary Material

Supplementary Figure 2

Supplementary Figure 1

Supplementary Table 1

Supplementary material associated with this article can be found in the online version at doi:10.1016/j.ijrobp.2022.10.033.
