# Supplementary material for: Prevention of Radiation-Induced Bladder Injury: A Murine Study Using Captopril
Source: Int J Radiat Oncol Biol Phys. Author manuscript; Available in PMC 2025 Jul 1. (PMC12210348; doi:10.1016/j.ijrobp.2022.10.033)
Supplement: Supplementary Figure 2 [file NIHMS2089868-supplement-Supplementary_Figure_2.pdf]

Supplementary Figure 1

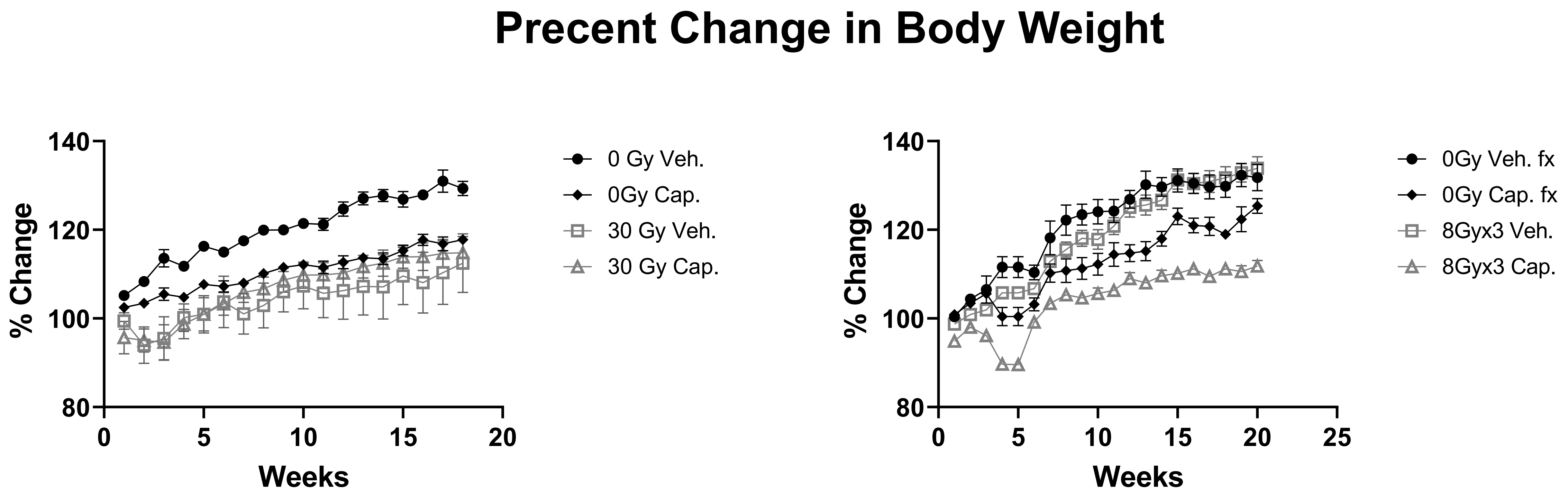

Legend: The 30 Gy single dose treatment caused a temporary reduction in weight which recovered by 5 weeks post irradiation and age-related weight gain was then evident. Captopril treatment did not limit age-related weight gain it did cause weight loss over the first two weeks of treatment.
