# Supplementary material for: Prevention of Radiation-Induced Bladder Injury: A Murine Study Using Captopril
Source: Int J Radiat Oncol Biol Phys. Author manuscript; Available in PMC 2025 Jul 1. (PMC12210348; doi:10.1016/j.ijrobp.2022.10.033)
Supplement: Supplementary Figure 1 [file NIHMS2089868-supplement-Supplementary_Figure_1.pdf]

Supplementary Figure 2

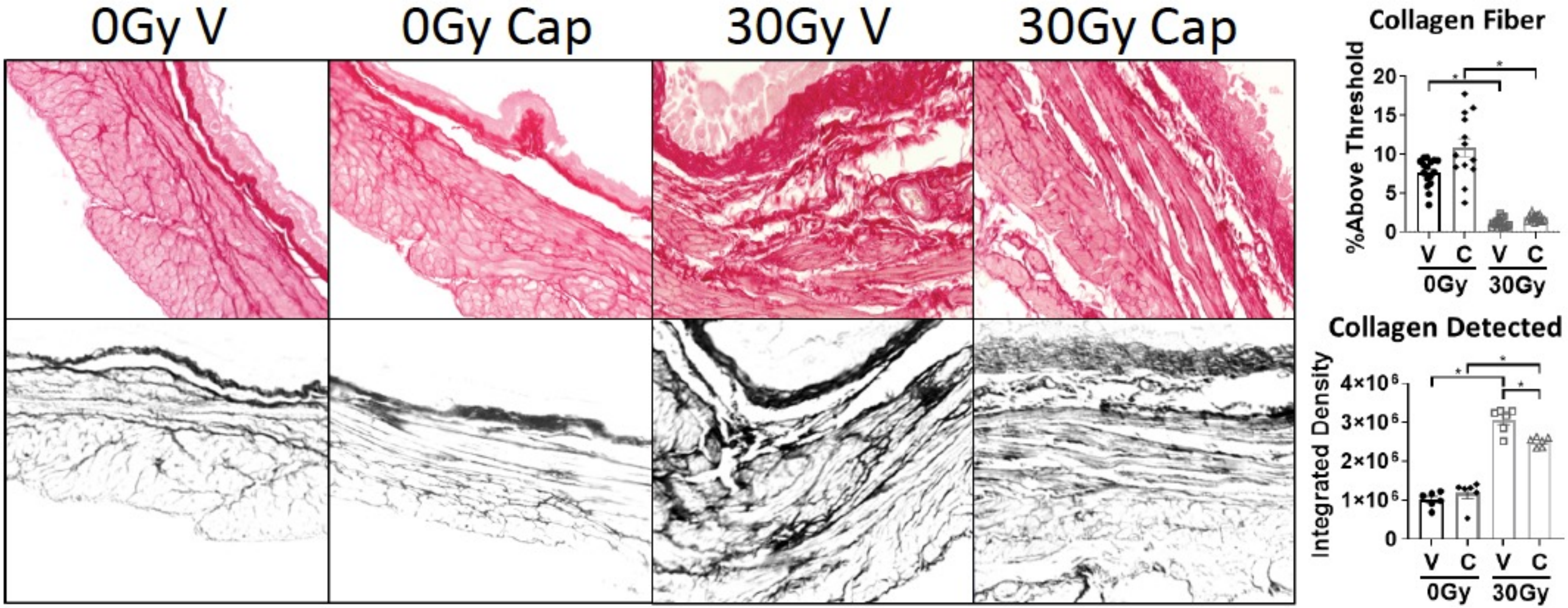

Quantification of collagen fiber integrity and density. Each Picro Sirius Red stained specimen was imaged in at least 3 fields, each with and without polarized light and brightfield images were acquired. For collagen fiber analysis, ImagePro Plus software was used to threshold pixels above a designated intensity and determine percentage above threshold. FIJI was used to measure integrated density of the threshold images. The images demonstrate loss of linear collagen organization and collagen deposition after 30Gy. Treatment with Captopril spared the loss of linear collagen organization compared with vehicle treatment.
