# Supplementary material for: Prevention of Radiation-Induced Bladder Injury: A Murine Study Using Captopril
Source: Int J Radiat Oncol Biol Phys. Author manuscript; Available in PMC 2025 Jul 1. (PMC12210348; doi:10.1016/j.ijrobp.2022.10.033)
Supplement: Supplementary Table 1 [file NIHMS2089868-supplement-Supplementary_Table_1.pdf]

Supplementary Table 1. Results of pathways enrichment analysis for differentially expressed genes in murine bladder tissue comparing 1 week post-30Gy + vehicle to 0Gy + vehicle, 1 week post-30Gy + captopril to 0Gy + vehicle, and 1 week post-30Gy + captopril to 30Gy vehicle. Inputs were differentially expressed genes at p-value < 0.05.

		1wk 30Gy vehicle vs 0Gy vehicle (5,573 genes input)		1wk 30Gy captopril vs 0Gy captopril (4,518 genes input)		1wk 30Gy captopril vs 30Gy vehicle (401 genes input)	
Pathway	Genes	Overlap	Adjusted P-value	Overlap	Adjusted P-value	Overlap	Adjusted P-value
Oxidative Phosphorylation	COX7B;SLC25A3;ACAA2;ALAS1;ECI1;MRPS11;COX4I1;MRPS12;ETFB;TCIRG1;MRPL34;COX6A1;PHB2;COX7C;TOMM22;CASP7;OPA1;ATP6V1E1;COX8A;BCKDHA;ATP6V0B;ATP6AP1;GPX4;ECH1;SDHC;NDUFC1;SDHB;COX6B1;AFG3L2;OXA1L;COX7A2L;BDH2;NDUFS8;NDUFS7;FDX1;UQCRC1;VDAC3;NDUFS3;VDAC2;NDUFS2;SUCLG1;SLC25A5;ATP6V0C;SLC25A11;ISCU;NDUFB8;OAT;NDUFB7;RETSAT;NDUFB6;ABCB7;NDUFB5;MGST3;NDUFB4;TIMM13;HTRA2;UQCR11;COX7A2;MRPL15;UQCR10;COX5B;COX5A;MRPL11;TIMM50;PRDX3;LDHB;LDHA;CYB5R3;GRPEL1;POLR2F;CYC1;SLC25A20;NDUFV2;NDUFV1;ATP6V1F;NDUFA8;CYB5A;SURF1;NDUFA7;NDUFA6;MDH2;NDUFA4;IDH3G;IDH1;NDUFA3;GOT2;NDUFA2;NDUFA1;COX6C;ACADSB;CS;ALDH6A1;UQCRQ;BAX;ACO2	95/200	1.21E-07	76/200	1.91E-05	2/200	0.952
p53 Pathway	CDKN1A;BTG2;CD81;CCP110;TRIAP1;IFI30;SAT1;RRP8;SEC61A1;HINT1;CCND3;GM2A;FAM162A;RPL36;KIF13B;RACK1;UPP1;IER5;HRAS;CTSD;IER3;PHLDA3;GPX2;PRMT2;SPHK1;DCXR;ABCC5;DNTTIP2;TAP1;AEN;FOS;PRKAB1;TM4SF1;VAMP8;PROCR;TRAF4;NINJ1;TAX1BP3;ISCU;EPHA2;PPP1R15A;PCNA;RETSAT;ABHD4;PVT1;HSPA4L;AK1;PDGFA;RPS27L;NOL8;SLC3A2;ACVR1B;KLK8;NDRG1;LDHB;SOCS1;RAB40C;HMOX1;SFN;BAK1;RPL18;RALGDS;SLC19A2;PLK3;JUN;ST14;CDKN2B;PMM1;PLK2;FUCA1;ZBTB16;EPHX1;OSGIN1;LIF;WWP1;KLF4;BLCAP;DEF6;BMP2;TCN2;KRT17;SP1;CCNG1;TNFSF9;FAS;BAX;MXD1;MXD4	88/200	1.71E-05	68/200	0.002	7/200	0.274
DNA Repair	CDA;MPG;GTF2B;SEC61A1;ZNRD1;CLP1;GUK1;REV3L;POLL;TK2;NELFE;RFC5;RFC3;GPX4;RFC2;VPS37D;NME4;EDF1;VPS37B;APRT;BCAM;RRM2B;DAD1;NFX1;BOLA2;POLR1D;SRSF6;EIF1B;VPS28;CCNO;TSG101;PCNA;CANT1;AK1;AAAS;ADCY6;NT5C;POLD4;POLR2C;POLD1;POLR2D;POLR2E;POLR2F;POLR2G;STX3;POLR2I;POLR2J;RAE1;SAC3D1;SURF1;DUT;BRF2;TAF10;ADRM1;UPF3B;RPA2;SMAD5;ZWINT;TARBP2;NUDT9;POLA1;NUDT21;POLA2;AGO4;ERCC1;IMPDH2;ERCC2;TAF6	68/150	5.77E-05	49/150	0.016	1/150	0.953
TNF-alpha Signaling via NF-kB	BTG2;CDKN1A;CD83;CSF1;TNFAIP6;CD80;TNFAIP2;LITAF;AREG;SAT1;SLC2A6;ICAM1;PANX1;ZFP36;CCND1;MYC;KYNU;SLC16A6;MAP3K8;CCN1;CCNL1;JUNB;IER5;IER2;IER3;DUSP4;MAP2K3;DUSP5;DUSP1;IFNGR2;SPHK1;PLAUR;TAP1;FOS;F3;RHOB;TUBB2A;IL1B;IRF1;NINJ1;SIK1;TRIB1;SGK1;FJX1;SQSTM1;PPP1R15A;NFAT5;CEBPB;SDC4;CEBPD;PTGS2;TANK;RELA;SOCS3;NFIL3;PDLIM5;MCL1;ABCA1;JUN;GADD45B;TIPARP;PLK2;LIF;ZBTB10;G0S2;CFLAR;ATP2B1;KLF4;PNRC1;KLF2;EIF1;VEGFA;NFKB2;FOSL1;EHD1;NR4A2;NR4A1;RCAN1;YRDC;BMP2;TNIP1;MAFF;TRIP10;TNFSF9;MXD1	85/200	7.15E-05	53/200	0.300	19/200	1.12E-06

Myc Targets V1	YWHAE;EIF4A1;SLC25A3;RPL34;RPLP0;HNRNPU;HNRNPR;PHB2;RPL6;UBE2L3;RRP9;PSMD8;SYNCRIP;PSMD7;XPO1;SNRPD2;TRIM28;YWHAQ;MYC;C1QBP;RUVBL2;PSMD3;RACK1;TXNL4A;CCT3;CCT2;SMARCC1;RPS5;IFRD1;RPS6;MRPS18B;TUFM;PSMA2;VDAC3;NHP2;PABPC1;PPIA;SNRPA;PCNA;CSTF2;SRSF1;CUL1;PHB;COX5A;PSMA7;SRM;PPM1G;PRDX3;LDHA;PSMB2;PSMB3;POLD2;RPS3;RPL14;EIF4H;RPS2;CYC1;BUB3;RPL18;CCT7;HNRNPA1;DUT;HNRNPA3;GOT2;PA2G4;DEK;HSPE1;LSM2;AIMP2;LSM7;PSMC4;CDK4;APEX1;IMPDH2;HNRNPA2B1;PRPF31;HDGF;TARDBP;ABCE1;EIF3D;EIF4G2;RAN;EIF3B	83/200	2.13E-04	64/200	0.012	4/200	0.857
Adipogenesis	SLC25A1;COX7B;ACAA2;SOWAHC;GBE1;HSPB8;ETFB;COX6A1;CPT2;DBT;ACADS;PGM1;COX8A;BCKDHA;JAGN1;GPX4;GPX3;MCCC1;ECH1;TALDO1;ELMOD3;LIFR;SDHC;SDHB;ACOX1;RAB34;UQCRC1;NDUFS3;ITGA7;SUCLG1;PREB;SLC25A10;ANGPTL4;ALDOA;TKT;PPP1R15B;CD151;NDUFB7;RETSAT;MGST3;LPL;ABCB8;UQCR11;MRPL15;UQCR10;TANK;ADIPOR2;ADCY6;AGPAT3;PRDX3;SAMM50;GRPEL1;DDT;DHRS7B;UCP2;SNCG;CYC1;DRAM2;RREB1;ABCA1;PTCD3;FZD4;MDH2;IDH3G;IDH1;VEGFB;CHCHD10;FAH;PEX14;SOD1;CS;COQ3;GPAM;UQCRQ;LPCAT3;PDCD4;NABP1;ACO2;CRAT	79/200	0.002	70/200	6.75E-04	3/200	0.857
Apoptosis	IFITM3;CDKN1A;BTG2;CDKN1B;HSPB1;CLU;SAT1;LGALS3;CASP7;CCND1;TNFSF10;TSPO;CASP2;TIMP1;DNM1L;IER3;GPX1;TNFRSF12A;GPX4;GPX3;HGF;MMP2;PPP2R5B;TAP1;CDC25B;EREG;RHOB;DNAJC3;IL1B;IRF1;DPYD;RARA;VDAC2;SQSTM1;RNASEL;RETSAT;ROCK1;SATB1;XIAP;NEDD9;RELA;EBP;ERBB3;LMNA;PMAIP1;HMOX1;BMF;CD14;MCL1;JUN;CREBBP;GADD45B;MGMT;GSR;CFLAR;SOD1;ISG20;CYLD;BMP2;KRT18;PDCD4;BAX;FAS;BCL2L1	64/161	0.005	46/161	0.132	5/161	0.464
Reactive Oxygen Species Pathway	PRNP;EGLN2;NDUFA6;SRXN1;GPX4;GPX3;NDUFB4;GSR;GLRX;IPCEF1;ATOX1;SOD1;PDLIM1;PTPA;PRDX2;MSRA;FES;STK25;PRDX1;ERCC2;NDUFS2;JUNB;HMOX2;LAMTOR5	24/49	0.008	18/49	0.073	1/49	0.857
UV Response Up	BTG2;ALAS1;TFRC;CLTB;HNRNPU;ARRB2;ICAM1;CCND3;HTR7;SPR;UROD;PPAT;HYAL2;BSG;PRKACA;JUNB;STARD3;PARP2;GPX3;IGFBP2;SIGMAR1;TAP1;FOS;TUBA4A;RHOB;OLFM1;CDC34;COL2A1;IRF1;SELENOW;TMBIM6;NPTXR;ALDOA;SQSTM1;CNP;MAOA;HSPA13;RXRB;DNAJB1;GRPEL1;AP2S1;HMOX1;MGAT1;BAK1;ATP6V1F;CYB5B;KLHDC3;CDKN2B;EPHX1;RAB27A;HSPA2;PTPRD;NR4A1;BMP2;EIF5;PSMC3;STK25;AGO2;FGF18;CYP1A1;GRINA	61/158	0.012	63/158	1.91E-05	5/158	0.464
Unfolded Protein Response	EIF4A1;CEBPB;POP4;EIF4A3;SDAD1;RRP9;CKS1B;RPS14;EXOSC5;TATDN2;EXOSC4;BAG3;KIF5B;EIF4EBP1;MTREX;BANF1;EXOSC1;TTC37;XBP1;IMP3;WFS1;FUS;LSM1;DDX10;LSM4;YWHAZ;YIF1A;VEGFA;ERN1;CNOT4;DNAJC3;CNOT6;TUBB2A;SPCS1;DKC1;CNOT2;NHP2;SRPRB;NABP1;PREB;CALR;ATP6V0D1;ATF6;DCP2;ATF4	45/113	0.018	31/113	0.347	2/113	0.857
IL-2/STAT5 Signaling	IFITM3;NRP1;PHTF2;ECM1;RNH1;CD83;GUCY1B1;BMPR2;CSF1;CD81;CTSZ;RORA;IKZF2;HK2;CCND3;ALCAM;GLIPR2;MYC;TNFSF10;PIM1;ENPP1;MAP3K8;TGM2;CISH;GPX4;GSTO1;S100A1;NCOA3;PUS1;APLP1;FAM126B;TNFRSF1B;ETV4;RHOB;TIAM1;TWSG1;MAPKAPK2;IL3RA;ITGA6;IRF6;PLPP1;NOP2;CAPG;NDRG1;AGER;SOCS2;NT5E;MUC1;SYNGR2;SOCS1;NFIL3;MAP6;GBP4;PRNP;XBP1;RABGAP1L;POU2F1;GADD45	73/199	0.018	57/199	0.103	11/199	0.015

	B;PRAF2;SPRY4;LIF;HUWE1;FAH;HOPX;BATF;TTC39B;BMP2;SNX14;MAFF;MXD1;EEF1AKMT1;F2RL2;BCL2L1						
PI3K/AKT/mTOR Signaling	GSK3B;CDKN1A;ARF1;CDKN1B;RALB;CAB39L;CLTC;PTEN;PRKAG1;SLC2A1;PIK3R3;DAPP1;ECSIT;ACACA;RPS6KA3;GRK2;PPP2R1B;ARHGDIA;CFL1;AKT1;SFN;RAC1;HRAS;AP2M1;MAP2K3;VAV3;ACTR2;PLA2G12A;TNFRSF1A;PPP1CA;TIAM1;PIKFYVE;CDK4;ARPC3;CSNK2B;AKT1S1;PIN1;CALR;PFN1;SQSTM1;MYD88;NFKBIB	42/105	0.020	37/105	0.013	2/105	0.857
Mitotic Spindle	YWHAE;DOCK4;NUMA1;SMC3;ARHGAP5;PKD2;SMC4;UXT;AKAP13;PCM1;OPHN1;PPP4R2;CAPZB;KIF5B;ARHGDIA;CDC27;CNTRL;TLK1;KIF1B;ARHGEF12;RFC1;MAP1S;CEP131;KIF23;TSC1;SMC1A;DYNLL2;CKAP5;TUBA4A;CD2AP;LATS1;TIAM1;ALS2;RASA1;ARHGEF3;CDC42EP2;FSCN1;CDC42EP1;KIF20B;SOS1;ARL8A;ARF6;PAFAH1B1;ROCK1;NEDD9;KATNB1;RASAL2;ABR;FGD4;CYTH2;FGD6;CEP192;RICTOR;PCNT;PDLIM5;SPTBN1;SAC3D1;ARFGEF1;MID1IP1;TBCD;HOOK3;CCDC88A;DLG1;NIN;APC;CENPJ;ABI1;NF1;TUBGCP5;MAP3K11;BCAR1	71/199	0.036	48/199	0.546	3/199	0.857
Hypoxia	SLC25A1;CDKN1A;CDKN1B;CITED2;GBE1;HEXA;SLC2A1;ADM;RORA;ENO1;VLDLR;ETS1;HK2;ZFP36;FAM162A;STBD1;PIM1;CCN2;CCN1;PPFIA4;PGM1;IER3;TGM2;TPI1;DUSP1;GAA;PGAM2;PLAUR;MIF;FOS;F3;JMJD6;SAP30;SULT2B1;TPST2;ILVBL;ANGPTL4;ALDOA;GAPDH;PPP1R15A;KDM3A;LXN;SDC4;PDGFB;GLRX;NDRG1;LDHA;PRDX5;NFIL3;HAS1;GPC1;HMOX1;PPARGC1A;ANKZF1;JUN;MAP3K1;TIPARP;SIAH2;CAVIN3;B3GALT6;KLHL24;PNRC1;HS3ST1;VEGFA;ISG20;SLC6A6;EFNA3;NAGK;XPNPEP1;MAFF;GALK1	71/200	0.036	51/200	0.380	14/200	8.73E-04
Glycolysis	B4GALT2;GAL3ST1;CHPF;CITED2;XYLT2;ENO1;VLDLR;HS6ST2;HK2;GMPPB;FAM162A;GMPPA;QSOX1;PGLS;PPFIA4;IER3;IL13RA1;TPI1;GLCE;PGAM2;AKR1A1;TALDO1;SDHC;ALG1;MIF;PHKA2;SAP30;TPST1;PKM;HAX1;B3GNT3;SLC25A10;RBCK1;ANGPTL4;ALDOA;ALDH7A1;PPIA;MET;DSC2;TSTA3;GPR87;GLRX;NOL3;CLN6;PPP2CB;LDHA;NT5E;CHST12;GPC1;NDUFV3;ANKZF1;LHPP;CYB5A;SLC35A3;HOMER1;MDH2;B3GAT3;AGL;IDH1;GFPT1;GOT2;B3GALT6;MERTK;VEGFA;SOD1;ISG20;EXT2;EFNA3;GALE;PSMC4;GALK1	71/200	0.036	60/200	0.040	4/200	0.857
Myc Targets V2	PUS1;NOP2;PPAN;WDR74;NOC4L;TMEM97;PHB;PA2G4;HSPE1;IPO4;HK2;RRP9;SRM;TBRG4;AIMP2;EXOSC5;LAS1L;CDK4;MYC;GRWD1;PES1;MRTO4;FARSA;DCTPP1	24/58	0.057	16/58	0.395	1/58	0.857
UV Response Dn	PHF3;NRP1;CDKN1B;CITED2;CELF2;PTEN;FHL2;VLDLR;RND3;RGS4;NIPBL;MYC;SLC22A18;CCN1;CDON;WDR37;IGFBP5;MIOS;DUSP1;ATRX;F3;VAV2;ATRN;DDAH1;KIT;MET;YTHDC1;GCNT1;PIK3R3;ATP2C1;ATXN1;SPOP;TOGARAM1;PDLIM5;MAP2K5;FZD2;BDNF;MGMT;NEK7;ATP2B4;NR1D2;SYNJ2;ATP2B1;PEX14;ACVR2A;DLG1;NFIB;PMP22;BMPR1A	49/144	0.178	29/144	0.857	3/144	0.857
Protein Secretion	NAPA;CD63;ARF1;TSG101;STX16;CLTC;SNAP23;CLTA;RPS6KA3;LMAN1;GOLGA4;AP1G1;AP2S1;KIF1B;DNM1L;AP2M1;SH3GL2;ARFGEF1;ABCA1;ARFGEF2;MON2;ADAM10;SCAMP1;SCAMP3;SOD1;VAMP7;KRT18;RER1;TMX1;DOP1A;GNAS;YIPF6;ERGIC3;COPE	34/96	0.178	35/96	0.012	1/96	0.915
Pperoxisome	SLC23A2;SLC35B2;ABCD2;ABCD3;RETSAT;CTBP1;MVP;ECI2;PEX11B;CLN6;HMGCL;PRDX5;ATXN1;PRDX1;LONP2;TSPO;MLYCD;HRAS;YWH	36/104	0.206	37/104	0.012	2/104	0.857

	AH;FIS1;SMARCC1;CADM1;IDH1;ABCC5;ECH1;ACSL4;DHCR24;PEX14;SOD1;SULT2B1;MSH2;ACOX1;EHHADH;ERCC1;PABPC1;CRAT						
mTORC1 Signaling	GSK3B;CDKN1A;BTG2;TFRC;GBE1;SLC2A1;TMEM97;ENO1;VLDLR;IFI30;HK2;RRP9;PSPH;PGM1;MAP2K3;ACTR2;TPI1;IGFBP5;IFRD1;ACSL3;TUBG1;TUBA4A;SYTL2;PSME3;ALDOA;SQSTM1;PPIA;GAPDH;PPP1R15A;TOMM40;SHMT2;PIK3R3;DAPP1;GLRX;CFP;CORO1A;ADIPOR2;ACACA;GGA2;SLC9A3R1;LDHA;EBP;PSMB5;NFIL3;PRDX1;ABCF2;CYB5B;XBP1;SDF2L1;HSPA4;NFYC;IDH1;GSR;DHCR24;HSPE1;MLLT11;SLC6A6;PSMC4;PSMC2;CCNG1;POLR3G;CD9;CALR;LGMN;NFKBIB	65/200	0.209	57/200	0.105	8/200	0.181
Cholesterol Homeostasis	MVK;LPL;TMEM97;CLU;CXCL16;LGALS3;EBP;ATXN2;ALCAM;NFIL3;FBXO6;S100A11;FDFT1;GSTM2;PCYT2;TNFRSF12A;ECH1;ANXA5;GPX8;PLAUR;PNRC1;ETHE1;CD9;MVD;ATF5;LGMN	26/74	0.248	20/74	0.395	4/74	0.196
Xenobiotic Metabolism	CDA;ABCD2;ALAS1;SLC35B1;PROS1;SPINT2;TMEM97;CROT;COMT;CNDP2;KYNU;UPP1;HES6;CBR1;ABCC2;GSTO1;IGFBP4;ENTPD5;DCXR;ECH1;DHPS;PGD;TNFRSF1A;ALDH3A1;TPST1;DDAH2;ACOX1;NINJ1;FETUB;BLVRB;TMBIM6;PTGES;EPHA2;RETSAT;MAOA;SHMT2;BPHL;PSMB10;TKFC;SERTAD1;DDT;HMOX1;ASL;PDLIM5;GSTM4;CCL25;CYB5A;PMM1;JUP;GSS;TMEM176B;IDH1;GSR;EPHX1;FAH;DHRS1;SLC6A6;PINK1;GSTA3;CYP1A1;FAS;ACO2;LPIN2;BCAR1	64/200	0.250	60/200	0.040	7/200	0.274
Interferon Alpha Response	IFITM3;LGALS3BP;IFITM1;SAMD9L;IFITM2;CSF1;CNP;GMPR;IFI35;IFI30;SLC25A28;MVB12A;TRIM25;TRIM26;B2M;GBP4;CD74;PNPT1;IL15;TAP1;IFI44;EIF2AK2;PARP14;PSMB8;BST2;ISG20;PROCR;ELF1;IRF1;PSME1;NCOA7;LY6E	32/97	0.337	30/97	0.112	5/97	0.181
Estrogen Response Early	LAD1;NXT1;MAST4;HSPB8;FHL2;SLC2A1;HR;AREG;PRSS23;CELSR2;RAPGEFL1;DEPTOR;CCND1;MYC;TBC1D30;JAK2;TGM2;TGIF2;CISH;IGFBP4;KRT8;FOS;RHOD;SULT2B1;TIAM1;AR;OLFM1;TUBB2B;ELF1;CLDN7;RARA;BLVRB;NBL1;PTGES;SLC26A2;CANT1;ASB13;TMEM164;SLC7A2;UGCG;SLC9A3R1;MUC1;INPP5F;ALDH3B1;P2RY2;NRIP1;RBBP8;PMAIP1;SFN;SLC19A2;FDFT1;XBP1;TIPARP;WFS1;SIAH2;KLF4;MED13L;KRT19;KRT18;SYT12;UNC119;FRK	62/200	0.346	43/200	0.817	11/200	0.015
Estrogen Response Late	NXT1;ST6GALNAC2;HSPB8;NAB2;HR;ETFB;PRSS23;AREG;CELSR2;CXCL14;RAPGEFL1;SLC2A8;ZFP36;CCND1;MDK;JAK2;UNC13B;CHST8;CISH;IGFBP4;DCXR;LSR;FOS;SULT2B1;TIAM1;OLFM1;DNAJC1;RABEP1;BLVRB;PKP3;SGK1;NBL1;PTGES;TSTA3;SLC26A2;RNASEH2A;HSPA4L;SCUBE2;SLC9A3R1;ALDH3B1;NRIP1;RBBP8;SFN;CKB;FDFT1;XBP1;ST14;HOMER2;WFS1;SIAH2;ATP2B4;COX6C;KLF4;ASS1;BATF;MAPK13;ISG20;KRT19;GALE;PDCD4;CD9;FRK	62/200	0.346	46/200	0.638	7/200	0.274
heme Metabolism	BTG2;NARF;TFRC;PIGQ;GDE1;SLC2A1;BACH1;ALAD;HEBP1;PSMD9;CCND3;UROD;BSG;CDC27;CIR1;PGLS;BTRC;FBXO7;RBM5;CTSB;HTATIP2;MAP2K3;ARHGEF12;KHNYN;GLRX5;PPP2R5B;RAD23A;VEZF1;GAPVD1;MINPP1;BCAM;DAAM1;TFDP2;BLVRB;TOP1;HAGH;OPTN;BLVRA;KDM7A;USP15;MGST3;HTRA2;ADIPOR1;PRDX2;UCP2;TSPAN5;SLC10A3;NEK7;XPO7;MBOAT2;PDZK1IP1;SLC25A37;ALDH6A1;SYNJ1;TMCC2;HDGF;LRP10;LPIN2;TMEM9B;NFE2L1;PICALM;TNRC6B	62/200	0.346	48/200	0.552	3/200	0.857
G2-M Checkpoint	JPT1;CDKN1B;CCNT1;NUMA1;HNRNPU;ARID4A;CTCF;SMC4;CKS1B;SYNCRIP;XPO1;PTTG1;CCND1;MYC;KIF5B;CDC27;CHMP1A;LBR;SMAR	61/200	0.402	50/200	0.395	3/200	0.857

	CC1;SLC38A1;ATRX;KIF23;PRPF4B;SMC1A;CDC25B;SAP30;ILF3;RBL1;EWSR1;DKC1;NCL;MTF2;TOP1;KIF20B;ATF5;PAFAH1B1;CUL5;YTHDC1;CUL3;SRSF1;CUL1;PDS5B;RASAL2;PURA;WRN;RAD21;DMD;E2F3;BUB3;E2F4;CASP8AP2;HSPA8;EGF;CBX1;HMGA1;RPA2;FOXN3;STAG1;POLA2;UBE2S;CDK4						
TGF-beta Signaling	PPP1R15A;BMPR2;SMURF2;IFNGR2;NOG;XIAP;ARID4B;TGFBR1;RHOA;PPP1CA;BMP2;APC;JUNB;SKIL;SPTBN1;TRIM33;ENG;BMPR1A	18/54	0.402	16/54	0.354	2/54	0.590
Inflammatory Response	IFITM1;CDKN1A;BTG2;CSF1;TNFAIP6;ADM;PVR;CX3CL1;ICAM1;MYC;TNFSF10;ITGB8;CCR7;KIF1B;TIMP1;SCN1B;IL15;IFNGR2;SPHK1;RHOG;PLAUR;EMP3;TNFRSF1B;F3;EREG;TAPBP;RNF144B;IL1B;IRF1;APLNR;MET;LY6E;C5AR1;NOD2;ACVR1B;ATP2C1;RELA;SLC7A2;P2RY2;PDPN;CD14;CCL17;KCNJ2;ABCA1;GABBR1;CCL22;FZD5;SLC31A1;ADRM1;LIF;EIF2AK2;OPRK1;ATP2B1;SELE;ACVR2A;BST2;ABI1;TNFSF9;MXD1	59/200	0.564	42/200	0.817	10/200	0.035
Androgen Response	SLC26A2;IQGAP2;SAT1;NDRG1;MYL12A;CDC14B;RPS6KA3;LMAN1;HERC3;CCND3;CCND1;AKT1;PDLIM5;B2M;HOMER2;TMEM50A;GSR;ADRM1;KRT8;ZBTB10;LIFR;DHCR24;ACSL3;PA2G4;MERTK;KRT19;APPBP2;VAPA;SGK1;PLPP1	30/100	0.587	27/100	0.377	3/100	0.623
E2F Targets	JPT1;CDKN1A;POP7;CDKN1B;TFRC;CCP110;PSIP1;SMC6;CTCF;SMC3;SMC4;IPO7;CKS1B;PNN;SYNCRIP;XPO1;PTTG1;MYC;LBR;RFC3;RFC1;RFC2;TUBG1;SMC1A;CDC25B;ILF3;MSH2;LUC7L3;NAA38;DCTPP1;SNRPB;PCNA;RNASEH2A;PRKDC;SRSF1;PDS5B;TBRG4;RAD21;POLD1;PPP1R8;POLD2;GINS1;DUT;CBX5;GINS4;ATAD2;HMGA1;RPA2;PA2G4;DEK;NUDT21;POLA2;STAG1;RAD50;UBE2S;CDK4;RAN	57/200	0.689	46/200	0.638	6/200	0.464
KRAS Signaling Up	NRP1;ADAMDEC1;PLVAP;CAB39L;CROT;ETS1;PRKG2;SCN1B;MAP4K1;ST6GAL1;FCER1G;PEG3;ITGA2;PLAUR;TNFRSF1B;ETV4;DUSP6;MMP10;EREG;ADAM17;IL1B;MALL;PECAM1;ANGPTL4;TRIB1;ENG;PPP1R15A;SATB1;GLRX;HOXD11;PTGS2;HDAC9;GPNMB;BTBD3;APOD;EVI5;CBX8;RABGAP1L;MAP3K1;PTCD2;JUP;FUCA1;TMEM176B;TMEM176A;LIF;LAPTM5;AMMECR1;G0S2;KLF4;GNG11;PSMB8;GADD45G;YRDC;BMP2;NIN;CCSER2;STRN	57/200	0.689	44/200	0.778	14/200	8.73E-04
Apical Surface	ATP8B1;PLAUR;HSPB1;ADAM10;LYPD3;THY1;SLC2A4;ADIPOR2;CX3CL1;PKHD1;SLC22A12;FLOT2;EPHB4	13/44	0.689	12/44	0.618	3/44	0.196
IL-6/JAK/STAT3 Signaling	ACVRL1;CSF1;ACVR1B;SOCS3;SOCS1;PIM1;HMOX1;MAP3K8;BAK1;CD14;IL13RA1;JUN;TNFRSF12A;IFNGR2;TNFRSF1B;TNFRSF1A;HAX1;IL1B;IRF1;IL3RA;FAS;CD9;LTBR;MYD88;CRLF2	25/87	0.689	20/87	0.666	7/87	0.015
Myogenesis	CDKN1A;ITGB5;HSPB8;HSPB2;CLU;SGCA;MB;BDKRB2;MYOZ1;PPFIA4;MEF2A;TEAD4;GABARAPL2;CHRNB1;PTGIS;ACTN3;ATP6AP1;GPX3;DAPK2;SPHK1;GAA;IFRD1;PGAM2;SIRT2;PTP4A3;MYL1;MYL2;APLNR;ITGA7;MAPRE3;KIFC3;CRYAB;MYH7;TAGLN;CTF1;AK1;FOXO4;SYNGR2;ERBB3;APOD;DMD;IGFBP7;CKB;PRNP;GADD45B;CAV3;AGL;SORBS3;SOD3;SMTN;DES;KLF5;PKIA;FXYD1;FKBP1B;CRAT	56/200	0.708	47/200	0.618	11/200	0.015
Interferon Gamma Response	IFITM3;LGALS3BP;CDKN1A;SAMD9L;IFITM2;TNFAIP6;SPPL2A;TNFAIP2;IFI35;IFI30;ICAM1;CASP7;TNFSF10;PIM1;TRIM25;TRIM26;ITGB7;UPP1;JAK2;B2M;IFNAR2;IL15;NCOA3;TAP1;TAPBP;VAMP8;LATS2;PSMA2;IRF1;PSME1;SERPING1;IRF5;RBCK1;LY6E;CD274;MVP;NLRC5;PTGS2;PS	56/200	0.708	42/200	0.817	10/200	0.035

	MB10;SOCS3;RNF213;SOCS1;SLC25A28;PSMB2;GBP4;CD74;PNPT1;LYSMD2;EIF2AK2;IFI44;PARP14;PSMB8;BST2;ISG20;FAS;MYD88						
Fatty Acid Metabolism	ACAA2;RETSAT;MAOA;ECI1;ECI2;ADIPOR2;BPHL;ALAD;HMGCL;LDHA;LGALS1;CPT2;UROD;PCBD1;MLYCD;ACADS;YWHAH;CBR3;CBR1;MDH2;IDH3G;IDH1;NCAPH2;ECH1;EPHX1;G0S2;SDHC;ACSL4;DHCR24;MIF;ALDH3A1;ACOX1;EHHADH;APEX1;ACOT2;PSME1;ERP29;CYP1A1;SUCLG1;ACO2;PPARA;ALDOA;CRAT;BLVRA	44/158	0.719	46/158	0.111	2/158	0.904
Apical Junction	CTNND1;ICAM2;PTEN;ACTB;CX3CL1;ICAM1;TIAL1;ADAMTS5;CDH3;MDK;RAC2;NRTN;HRAS;YWHAH;MAP4K2;ACTN3;ITGA2;MMP2;TSC1;TUBG1;VAV2;CLDN5;ADAM15;RASA1;CLDN7;FSCN1;CERCAM;PECAM1;MVD;PFN1;MYL9;CD274;PIK3R3;NEXN;THY1;MYL12B;GNAI2;CNN2;LIMA1;RRAS;NRAP;TSPAN4;VASP;JUP;MPP5;SORBS3;MAPK13;DLG1;ARPC2;ZYX;NF1;CRAT;NECTIN2;CRB3	54/200	0.813	50/200	0.395	3/200	0.857
Complement	CDA;DOCK4;GMFB;DYRK2;DOCK9;CLU;LGALS3;CASP7;PCLO;GNGT2;CSRP1;KYNU;PIM1;CTSH;TIMP1;JAK2;CTSD;CTSB;DUSP5;FCER1G;ANXA5;RHOG;PLAUR;SIRT6;F3;RBSN;DUSP6;F8;PIK3CA;IRF1;FDX1;SERPING1;PFN1;DGKH;USP15;CEBPB;USP16;PDGFB;GNAI2;PPP2CB;VCPIP1;PPP4C;S100A13;RCE1;PRSS36;ATOX1;EHD1;XPNPEP1;GNB2;MAFF;CALM3;LGMN;C1QC;MSRB1	54/200	0.813	43/200	0.817	7/200	0.274
Notch Signaling	APH1A;PSENEN;MAML2;CCND1;FZD5;CUL1;DTX2;SAP30	8/32	0.864	10/32	0.377	1/32	0.857
Hedgehog Signaling	NRP1;LDB1;OPHN1;RASA1;NF1;THY1;VLDLR;AMOT;VEGFA	9/36	0.864	9/36	0.629	NA	NA
Coagulation	PEF1;PROS1;HTRA1;PDGFB;CRIP2;CLU;KLK8;PRSS23;CPN1;CSRP1;P2RY1;S100A13;CTSH;TIMP1;RAC1;CTSB;S100A1;MMP2;ITGA2;MST1;MMP3;F3;MMP10;SIRT2;DUSP6;F8;GNB2;MAFF;PECAM1;SERPING1;CD9;ISCU;RAPGEF3;F2RL2;LGMN	35/138	0.920	20/138	0.997	5/138	0.347
Bile Acid Metabolism	SLC35B2;SLC23A2;ABCD2;ABCD3;RETSAT;CROT;TFCP2L1;PRDX5;ATXN1;SLC22A18;LONP2;MLYCD;NUDT12;ABCA1;PEX19;ABCA5;PNPLA8;IDH1;DIO2;DHCR24;PEX1;CYP7B1;SOD1;SULT2B1;AR;RBP1;OPTN	27/112	0.977	30/112	0.377	2/112	0.857
Epithelial Mesenchymal Transition	ECM1;SERPINE2;ITGB5;PLOD3;AREG;SAT1;PVR;RGS4;GLIPR1;EFEMP2;LGALS1;QSOX1;CCN2;TIMP1;CCN1;TGM2;TNFRSF12A;IL15;IGFBP4;MMP2;ITGA2;IGFBP2;APLP1;MMP3;PLAUR;GPX7;EMP3;RHOB;ACTA2;PPIB;MYL9;TAGLN;SDC4;LAMA1;HTRA1;CAPG;THY1;PTHLH;NT5E;GPC1;JUN;GADD45B;CADM1;BDNF;FUCA1;VEGFA;PMP22;FAS;SNTB1	49/200	0.994	46/200	0.638	11/200	0.015
Angiogenesis	NRP1;LPL;PDGFA;TIMP1;PGLYRP1;VEGFA;VAV2	7/36	1.000	7/36	0.817	NA	NA
Allograft Rejection	CSF1;CD80;GCNT1;CAPG;MTIF2;THY1;CFP;RPL9;ETS1;HDAC9;PSMB10;ICAM1;CCND3;SOCS1;RPS19;AKT1;CSK;TIMP1;APBB1;JAK2;B2M;RPL39;MAP4K1;IFNAR2;EIF5A;CD74;CD96;RPS9;CCL22;IL15;IFNGR2;LIF;TAP1;ACVR2A;EREG;TAPBP;IL1B;ABI1;FAS;KLRD1;DEGS1;ABCE1;EIF3D;EIF3A	44/200	1.000	42/200	0.817	4/200	0.857
Wnt-beta Catenin Signaling	WNT6;ADAM17;WNT5B;MYC;CUL1	5/42	1.000	12/42	0.395	1/42	0.857
Pancreas Beta Cells	SPCS1;SRPRB;SRP9	3/40	1.000	3/40	0.997	1/40	0.857
KRAS Signaling Dn	RYR2;BTG2;THRB;PLAG1;CD80;ARHGDIG;LYPD3;TFCP2L1;KLK8;CELSR2;TGM1;MFSD6;NRIP2;SLC16A7;SKIL;KRT4;DTNB;SPHK2;EGF;ZBTB1	34/200	1.000	40/200	0.886	9/200	0.087

	6;IGFBP2;COPZ2;GPR3;THNSL2;DLK2;NR4A2;FGF16;COL2A1;NR6A1;STAG3;KLHDC8A;SIDT1;SGK1;MYH7						
Spermatogenesis	PGS1;MLLT10;PARP2;STRBP;HSPA4L;VDAC3;PEBP1;TALDO1;BRAF;HSPA2;IL13RA2;PIAS2	12/135	1.000	23/135	0.994	1/135	0.953
